# Implementation and evaluation of a technical support center for the Research to Enhance the Adaptation and Implementation of Health Systems Guidelines in LMICs [RAISE] initiative

**DOI:** 10.1186/s12961-026-01462-9

**Published:** 2026-09-10

**Authors:** Christine Fahim, Joshua Pratt, Amber Purewal, Leah Marsot-Shiffman, Keelia Quinn de Launay, Richelle Baddeliyanage, Larkin Davenport-Huyer, Sonam Yangchen, Azmach Hadush Gebregiorgis, Charles S. Wiysonge, Andrea C. Tricco, Lisa Puchalski-Ritchie, Ba’ Pham, Ozge Tuncalp, Etienne V. Langlois, Robert Marten, Sharon E. Straus

**Affiliations:** 1 Li Ka Shing Knowledge Institute, St. Michael’s Hospital, Unity Health Toronto, 209 Victoria Street, Toronto, ON M5B 1T8 Canada; 2 Dalla Lana School of Public Health, University of Toronto, 155 College St, Toronto, ON M5T 3M7 Canada; 3 Institue of Health Partners, CJMM+45H, PO Box 1435, Thimbu, Bhutan; 4 World Health Organization Regional Office, Plot 4609 Corner Andrew Mwenya/Beit Road, Roadspark, PO Box 32346, Lusaka, Zambia; 5 Cochrane South Africa, South African Medical Research Council, Francie Van Zijl Drive, Parow Valley, Tygerberg, PO Box 19070, Cape Town, 7505 South Africa; 6 HIV and Other Infectious Diseases Research Unit, South African Medical Research Council, 491 Peter Mokaba Ridge Road, Overport, Durban, South Africa; 7 Queen’s Collaboration for Health Care Quality: A JBI Centre of Excellence, Queen’s University, 92 Barrie Street, Kingston, ON K7L 3N6 Canada; 8 Department of Medicine, University of Toronto, 1 King’s College Circle, Toronto, ON M5S 1A8 Canada; 9 Department of Reproductive Health and Research, UNDP/UNFPA/UNICEF/WHO/World Bank Special Programme of Research, Development and Research Training in Human Production [HRP], World Health Organization, Avenue Appia 20, 1211 Geneva, Switzerland; 10 Partnership for Maternal, Newborn and Child Health [PMNCH], World Health Organization [WHO], Avenue Appia 20, 1211 Geneva, Switzerland; 11 Alliance for Health Policy and Systems Research, World Health Organization, Avenue Appia 20, 1211 Geneva, Switzerland

**Keywords:** Knowledge translation, Capacity strengthening, Implementation, Support, LMIC, Health policy and systems research

## Abstract

**Background:**

Capacity-strengthening initiatives for health policy and systems research (HPSR) in low- and middle- income countries (LMICs) are often designed with little consideration of LMIC stakeholder needs. We formed a Technical Support Centre to support research teams through the WHO Alliance for HPSR’s Research to Enhance the Adaptation and Implementation of Health Systems Guidelines (RAISE) initiative. We describe the implementation and evaluation of the support program.

**Methods:**

The support program was rooted in an integrated knowledge translation approach, whereby RAISE teams informed its design, content and delivery. Using mixed methods, we assessed quality of program implementation and its impact on RAISE teams’ knowledge and capacity for HPSR and implementation research. Data were collected between August 2019 and November 2021.

**Results:**

A total of 22 participants from six countries completed a needs assessment survey to inform design of the support program. The program included: an in-person capacity-strengthening workshop, 1:1 coaching with methods experts and virtual webinar series, discussion boards and workshops. RAISE teams perceived the supports to be of high quality. Mean scores for 8/9 research capacity outcomes improved over time; significant differences were observed in researchers’ ability to develop evidence-based knowledge translation interventions (*Z* = −2.93, *p* < 0.05). Barriers to health system guideline adaptation and implementation included competing health system and stakeholder interests, lack of available data and equipment to facilitate data collection and lack of knowledge and skills among those tasked with implementing the guideline. Enablers included partnering with health system stakeholders at project inception, employing a multi-sectoral approach to implementation and building the competencies and skills of the implementation team.

**Conclusions:**

Participants found the capacity strengthening program to be of high quality; participants’ research capacity and skills improved over time and significant differences in the ability to develop evidence-based knowledge translation interventions were observed. Despite barriers experienced, particularly in the context of the COVID-19 pandemic, RAISE teams reported that the support program strengthened their knowledge and supported them to engage diverse stakeholders in their research. Ongoing efforts to sustain capacity strengthening for health systems and HPSR implementation in LMICs are warranted.

*Registration*: doi.org/10.17605/OSF.IO/EF43J.

**Supplementary Information:**

The online version contains supplementary material available at https://doi.org/10.1186/s12961-026-01462-9.

## Background

Health policy and systems research (HPSR) is an interdisciplinary field that aims to enhance and evaluate how health systems and actors shape and implement health policies [1, 2]. HPSR is critical to advancing the United Nations’ Sustainable Development Goals to improve health systems using high-quality evidence implementation [3]. Investments in research on health system guideline optimization are one key focus on HPSR; health systems guidelines aim to improve health systems performance by addressing health systems challenges via the implementation, monitoring and evaluation of relevant strategies [4, 5]. Unlike clinical practice guidelines, which focus on providing evidence-based care to individual patients, health system guidelines have the capacity to optimize the efficiency, equity and quality of patient care at a system level [6]. Despite their potential impact, there is a gap in the availability and quality of health systems guidelines designed in Low and Middle Income Countries (LMICs) [6]. As such, health system stakeholders in LMICs often rely on guidelines developed by international bodies, such as the World Health Organization (WHO), or by high income countries [6–10]. Many of these guidelines are not adapted to the needs and context of LMICs, nor are they aligned with the priorities of policymakers and health system actors, posing a significant implementation challenge [9, 11]. Further, there is a paucity of evidence related to adaptation of health systems guidelines in LMICs, suggesting a need for research describing strategies to adapt and implement these guidelines in these settings [9, 12].

Capacity strengthening initiatives may close this gap. LMIC researchers are in the best position to identify health system needs and guide health policymakers to implement evidence that is culturally, contextually and politically aligned with stakeholder priorities [13]. To support capacity strengthening among LMIC researchers, the WHO Alliance for HPSR (the Alliance) created the RAISE (Research to Enhance the Adaptation and Implementation of Health Systems Guidelines in low- and middle- income countries (LMICs)) portfolio. The Alliance aims to improve health in LMICs by enhancing capacity for, and use of, HPSR [2, 4]. Through the RAISE portfolio, the Alliance funded six research teams from LMICs to advance the science on health systems guideline adaptation and implementation and the uptake of guidelines in LMIC settings [9, 14]. Another key feature of the RAISE portfolio was prioritization of capacity building in HPSR among key stakeholders, which included both researchers and policymakers. Researchers applying to the RAISE portfolio were required to partner with at least one principal applicant policymaker or knowledge user (defined as an individual who is likely to be able to use research results to make informed decisions), to support practical uptake of the guidelines [15].

To support the RAISE teams, the Alliance also formed a Technical Support Centre tasked with strengthening research capacity among RAISE teams and providing ongoing scientific support for knowledge translation, HPSR and guideline adaptation and implementation methodology. Following a competitive process, the Alliance selected the Knowledge Translation Program at St. Michael’s Hospital-Unity Health Toronto, in partnership with known networks at the South African Cochrane Centre and the WHO Ethiopia country office, to act as the RAISE Technical Support Centre.

In this manuscript, we describe our process of co-developing, implementing and evaluating the Technical Support Centre. Our objectives were to: Describe the process of co-developing and delivering the support program [Phase 1]; Assess quality of the support program [Phase 2]; and Assess impact of the support program on RAISE teams’ knowledge and research capacity and describe barriers and enablers experienced by RAISE teams when adapting and implementing health systems guidelines [Phase 3].

## Methods

We submitted our study protocol to Open Science Framework on 13 December 2019 (https://doi.org/10.17605/OSF.IO/EF43J).

### Overview of RAISE teams

In partnership with the Alliance, the Technical Support Centre facilitated a peer review process for proposals submitted for funding. A total of 27 proposals were submitted in response to the RAISE call; of these, 21 proposals met the eligibility criteria and were sent for peer review [14]. Each proposal was reviewed by two researchers, including at least one from a LMIC and a knowledge user (namely, policymakers and health system managers). A total of 16 reviewers participated in the selection process. Reviewers scored proposals on a weighted scale of 0–100 that assessed team qualifications, quality of research methods, strategy to engage decision makers, scope for impact, innovation, timeline feasibility and value for money. Scores ranged from 45.00 to 90.67. Scores were submitted to an international adjudication panel, assembled by the Alliance. The panel selected six proposals, considering scores and geographic representation.

The six selected RAISE teams were diverse in geography and guideline focus. The teams included representation from: Ghana (development of home visit guidelines), Colombia (prioritization of WHO health system guidelines in the Colombian context), India (implementation of antenatal care guidelines in tribal communities), Zambia (implementation of cholera control guidelines), Nigeria (adaptation and implementation of the WHO digital intervention guideline for mental health system strengthening) and Mozambique (implementation of guidelines to reduce primary care waiting times). See Appendix A for a summary of RAISE teams’ projects.

### Overview of study phases

Development and implementation of the RAISE technical support program was rooted in integrated knowledge translation methodology, meaning knowledge users – namely RAISE teams including researchers, trainees and policymakers – were engaged in the design and implementation of the program. Integrated knowledge translation improves buy-in for implementation efforts, thereby increasing evidence uptake and reducing research waste [16]. RAISE teams were also encouraged to apply an integrated knowledge translation approach to their work.

This study was composed of three phases (Table 1). In Phase I, we conducted a needs assessment with the six country teams to inform the design of the support program. Participants were asked to describe the challenges, opportunities and learning gaps related to HPSR in their settings. In Phase II, we conducted a process evaluation to assess implementation quality of the support program. In Phase III, we describe the impact of the support program on RAISE teams’ self-efficacy to conduct Knowledge Translation and HPSR and describe teams’ experiences with implementing health systems guidelines in LMIC settings.

**Table 1:** Summary of project phases

Phase	Outcomes	Data collection method and participants	Theories, tools
1: Needs assessment and monitoring	-Identification of capacity strengthening needs	Interviews: Offered to RAISE team research leads and knowledge users [August 2019] Surveys: Offered to all RAISE team personnel [August–September 2019] Document review: RAISE project protocols [August 2019], monitoring templates [August 2019–August 2021]	• Integrated knowledge translation approach [16]
2: Process evaluation	-Reach -Participants’ responsiveness and satisfaction with the support program -Implementation quality of the support program	Surveys: Offered to RAISE team personnel [August 2019–July 2021] Interviews: Offered to RAISE researchers and knowledge users [July–November 2021] Document review: Monitoring templates [August 2019–August 2021]	• Durlak and Dupre process evaluation metrics [17]
3: Impact of the Technical Support Centre; experiences with guideline adaptation and implementation	-Self-efficacy of implementation methods, skills, knowledge -Research utilization and comfort with HPSR -Barriers and enablers to adapting and implementing health systems guidelines	Surveys: Offered to all RAISE team personnel [baseline: August 2019; 12 month survey: August 2020; 24 month survey August 2021] Exit interviews: Offered to RAISE researchers and knowledge users [July–November 2021]	• Validated 7-item self-efficacy tool [20] • Theoretical Domains Framework [21] • Consolidated Framework for Implementation Research [22]

*Phase I:* We conducted needs assessment interviews to identify topic recommendations for the capacity-strengthening sessions. Interviews were conducted by research staff who supported program delivery (K.Q.L., R.B.) in August 2019 with a principal investigator or knowledge user from each of the six RAISE teams. All RAISE research team personnel (e.g., research staff, trainees) were also invited to complete a needs assessment survey. Participants ranked the importance of a list of research topics (informed by the team’s project proposals and the needs assessment interviews) to inform the content for a mandatory inception workshop and subsequent virtual webinars to strengthen methods skills and knowledge. A document review of the RAISE teams’ project protocols was conducted by research staff to identify potential learning/methods gaps. In addition, RAISE teams were required to submit monitoring templates describing study and knowledge user engagement progress every 3 months; these were reviewed by research staff to iteratively refine the content of the support program and provide tailored supports to RAISE teams, as required.

*Phase II:* We conducted a process evaluation using the Durlak and Dupre process evaluation metrics [17]. *Reach* of the program was assessed by determining the number of RAISE team members that participated in the support activities and by tracking the number of support requests received by the Technical Support Centre over time. *Responsiveness* and *satisfaction* with the support program were assessed using online surveys following delivery of each support activity (e.g., online webinar). We administered one in person survey following the inception workshop in Istanbul, Turkey (September 2019). The remaining surveys were administered online using Qualtrics software [18]. Survey links were sent to participants via email or provided to participants on screen during live virtual events. Reminders to complete monitoring surveys to assess responsiveness and satisfaction were sent to participants, following the Dillman total design survey method [19]. *Implementation quality* was assessed using exit interviews with the RAISE teams. Exit interviews were conducted using a semi-structured approach by research staff who were not involved in the delivery of RAISE capacity building interventions (A.P., J.P.). Research staff aimed to recruit interview participants via email, aiming for representation by RAISE team and participant role (researcher vs knowledge user), though all team members were invited to participate.

*Phase III:* To evaluate impact of the support program, we assessed researchers’ self-efficacy scores via surveys at baseline (August 2019), 12-months (August 2020) and 24-months (August 2021). The survey included seven questions on self-efficacy in the practice of Knowledge Translation (these items were previously validated for face validity by 10 knowledge translation experts) [20] and two questions on research utilization and comfort using evidence. Each question presented a research activity (e.g., *I can conduct a needs assessment for knowledge users*) on an anchored Likert scale of 1–7 where 1 represented terrible (*I can not do this at all*) and 7 represented excellent (*I can do this well*). During the exit interviews, we asked participants about their perceptions of the support program and their experiences adapting and implementing health systems guidelines. Specifically, we aimed to identify barriers and enablers to implementing health systems guidelines in LMICs. The Theoretical Domains Framework (TDF) [21] and Consolidated Framework for Implementation Research (CFIR) [22] were used to develop the interview guide and probe for individual, contextual and systems barriers and enablers to guideline adaptation or implementation.

*Participants:* Participants included researchers, trainees and knowledge users from the six funded RAISE teams in Colombia, Ghana, India, Mozambique, Nigeria and Zambia. Participants were assigned a unique study ID and provided consent prior to beginning online surveys and interviews. The RAISE principal investigators consented to use of study documents for document review.

*Data Analysis:* Descriptive statistics were used to summarize survey scores for individual webinar feedback surveys and impact surveys; and were analysed using SPSS v2 [23]. A non-parametric two-sample Wilcoxon rank-sum test was used to determine whether there were statistically significant differences (*p* < 0.05) in outcomes between time points.

Needs assessment interview data were analysed using a rapid analysis approach to facilitate rapid development of the support program [24]. Exit interviews were audio-recorded and transcribed verbatim. Interviews were coded using a thematic analysis [24]. Two researchers independently read three transcripts to generate a codebook. This codebook was piloted on three transcripts and refined iteratively; 20% of the transcripts were double-coded, and inter-rater reliability using Cohen’s kappa was calculated. Discrepancies between –1 and 0.6 were discussed and resolved by a third reviewer. Qualitative analyses were conducted using QSR NVivo v11 [25]. Data were themed by two researchers.

*Ethics:* This study received ethics approval through the Unity Health Toronto Research Ethics Board #19-202.

## Results

### Phase I – development of the technical support centre

*Needs Assessment Findings*: A total of 22 individuals completed the needs assessment survey (*N* = 35, 63% response rate). Participants included 10 men (45%) and 12 women (55%). Participant roles included seven principal investigators, six co-investigators, two knowledge users and six research staff. All teams found it challenging to identify their specific methods needs, as they had not yet begun their projects or did not have a comprehensive overview of their planned adaptation or implementation processes. Six teams requested rigorous training on evidence-based methods for implementation. Other capacity-strengthening topic requests included barrier and facilitator assessments (*n* = 4 teams), optimizing stakeholder engagement (*n* = 4 teams) and qualitative analysis methods (*n* = 3 teams).

Survey participants suggested that the RAISE inception workshop focus on capacity strengthening on the use of Knowledge Translation and HPSR methods to guide health systems guideline implementation. With respect to the ongoing capacity-strengthening workshops, the topic most highly ranked was how to use knowledge translation principles for guideline implementation (Appendix B). Other requested topics included qualitative analysis for implementation research, and frameworks for monitoring and evaluation in HPSR research.

*Identification of support strategies:* Via feedback collected from the needs assessment, we identified the following strategies for the support model: using the mandatory inception workshop as an opportunity to foster cross-team collaboration and build research skills, hosting ongoing workshops and webinars and providing tailored coaching to support teams with their implementation efforts.

### Implementation of the Technical Support Centre strategies

*Inception Workshop:* The Technical Support Centre, in partnership with the Alliance, led a mandatory workshop to support teams to refine their research protocols and health system adaptation or implementation strategies. The principal co-investigator and a knowledge user from each RAISE team were invited by the Alliance to attend a 3-day in-person workshop in Istanbul, Turkey in September 2019. The workshop modules included: introduction to knowledge translation and HPSR, identifying health system gaps, assessing quality of guidelines [26, 27], prioritizing guideline recommendations using Delphi processes, adapting guidelines [28, 29], identifying implementation actors and the evidence-based practice, assessing barriers and enablers to change [21, 22], mapping barriers and enablers to implementation strategies, evaluating quality of implementation, and outcomes and ethical considerations in implementation research. Modules were delivered by methods experts (S.E.S., E.L., C.F., K.Q.L., A.H.); a facilitated discussion followed each module. RAISE teams also had the opportunity to present their projects and receive feedback from methods experts and other RAISE teams. Each day, RAISE teams conducted project work using a protocol template to iteratively revise their methods and objectives.

*Webinar Series:* Seven capacity-strengthening virtual webinars were delivered by the Technical Support Centre between November 2019 and January 2021. Webinars were pre-recorded by content experts and were available for on-demand viewing via Canvas, an online learning platform [30]. Education materials (e.g. worksheets, relevant literature) supplemented the webinars. Webinar topics included: introduction to knowledge translation and HPSR, use of the Appraisal of Guidelines for Research and Evaluation II (AGREE) approach to guideline development [26, 27], evaluation and adaptation, methods for guideline adaptation, engaging stakeholders in research, methods for conducting qualitative research (including rapid qualitative analysis), contextual considerations for implementation in LMIC settings and project management and administration. Two live webinars were held in April and September 2020 to provide teams an opportunity to share project updates and participate in a lessons learned discussion with other RAISE teams and content experts.

*Online Discussion Boards:* Teams had access to discussion boards on the Canvas platform [30] and were invited to use them to discuss common challenges, barriers, enablers and questions.

*Virtual Workshops:* The Technical Support Centre originally planned to host in-country workshops to build research capacity among RAISE research teams and trainees. Owing to the COVID-19 pandemic, the in-country visits were pivoted to virtual workshops. Non-RAISE team members (i.e., other researchers, trainees and knowledge users associated with the RAISE teams and their organizations) were also invited to participate in the virtual workshops. A needs assessment survey circulated to RAISE teams was conducted to inform the content for these workshops. The virtual workshops were conducted from April to July 2021 and consisted of the following activities:

1. Live Qualitative Research Webinar: Interactive webinar focused on working through practical qualitative data analysis examples to address teams’ request for guidance on analysing qualitative data using traditional and rapid approaches [24].
2. Knowledge Translation and HPSR Seminar Series: A four part online seminar series on: adapting and appraising guidelines, developing an implementation plan, planning for evaluation scale-up and spread, and project management and practical challenges to implementation. Each section included 2–3 pre-recorded content webinars and a live session presented and facilitated by a content expert.

*Ongoing communication and coaching calls:* The Technical Support Centre offered ongoing support to RAISE teams through email and phone calls throughout the project period. Methods experts (C.F., S.E.S.) led coaching calls.

### Phase II – Evaluating the quality of the support program

*Quality of the Inception Workshop:* A total of 11 RAISE team members (five principal/co-principal investigators, three researcher co-investigators, one knowledge user co-investigator and two research staff) attended the in-person workshop in September 2019. Ten participants (91% response rate) completed the feedback survey at the workshop conclusion. Participants were largely satisfied with the workshop (mean rating 6.30/7). Open-ended responses indicated that participants found the content and resources to be of high quality and applicable to their projects. Moreover, participants appreciated the opportunity to engage with the other teams and consult with the Technical Support Centre and the Alliance. Suggestions for improvement were to include more examples and interactive activities, more time to work on protocols and apply the frameworks and more interactive group work with other project teams (Table 2). Interview participants (*n* = 9) found the workshop to be helpful overall as it provided a baseline for the RAISE projects and fostered a sense of collaboration between RAISE teams.

**Table 2:** Summary of inception workshop feedback

Question*	Mean ± SD [*n* = 10]
I was extremely satisfied with the workshop readings and resources	6.67 ± 0.5
Overall, I was satisfied with the presentations	6.11 ± 1.27
Overall, I was satisfied with how the content applies to my work	6.67 ± 0.71
I was satisfied with the workshop activities	6.20 ± 1.23
I was satisfied with the format of the workshop [presentation, group activity etc.]	5.70 ± 1.83
I was satisfied with the content of the workshop	6.50 ± 0.53
Overall, I thought the workshop was implemented with high quality	6.60 ± 0.52

^*^Likert scale response options: 1 = strongly disagree, 4 = neither agree nor disagree, 7 = strongly agree

*Additional supports:* Two scientists from the Technical Support Centre (C.F., S.S.) provided scientific feedback on project protocols. The Technical Support Centre prepared WHO Research Ethics Review Committee submissions for the six teams. To support implementation of the RAISE teams’ guidelines, the support team provided seven coaching calls and ongoing email communications.

*Quality of the Webinar series:* Average Canvas page views were 9.33 for the pre-recorded webinars, 6.5 for the discussion boards and 2.82 for the additional resources. The *AGREE Approach to Guideline Development, Evaluation and Adaptation* webinar had the highest page views across Canvas webinars (*n* = 22), discussion boards (*n* = 15) and resource pages (*n* = 9) (Appendix C). The April 2020 live webinar, which consisted of an introduction to the Technical Support Centre and 6-month progress update presentations from each RAISE team, had the highest attendance of all webinars (*n* = 30).

Response rates for post-webinar evaluation surveys ranged from 15 to 50%. All seven webinars were rated highly, at 4–5 out of 5 (slightly *above average* to *outstanding*). Participants identified the structure and organization, clarity of presentation and relevancy of information as key strengths of the webinars.

Six co-investigators, two research coordinators and one principal investigator (*n* = 9) participated in the exit interviews between July-November 2021. Participants indicated that the webinars contributed to capacity development in adaptation and implementation research. They noted that the on-demand nature of pre-recorded webinars provided flexibility and optimized accessibility for research teams. The webinars about AGREE tools, stakeholder engagement, rapid analysis and project management were found to be the most useful topics.

*Quality of the Technical Support Centre:* Participants had a positive perception of the supports provided by the Technical Support Centre. Researchers stated that the centre was involved throughout the project duration and supported the planning, implementation and dissemination stages and believed the centre strengthened research capacity and skills. Participants appreciated the multiple modes of supports although some participants were overwhelmed with the number of supports and activities offered and had a difficult time finding time for both capacity strengthening and RAISE project work. For example, participants felt that the quarterly reporting requirements helped keep them accountable to project progress, but found this task time consuming and repetitive. One co-principal investigator noted:

*“I think, obviously, a lot of people were willing to attend the [capacity-strengthening] sessions, but I think just balancing that up with the actual research implementation was sort of a barrier and I do want to just state that I think most for those that were not attending, I don’t think it’s because they didn’t want to, but because I have to also do this other stuff. So, I think finding a way to sort of balance up implementation plus webinars, I think would be my recommendation to the team.” - P11*

Similarly, knowledge user participation in the evaluation study was limited due to turnover, heavy workload and limited availability during the pandemic. Overall, teams found the supports to be accessible and believed they provided a mentorship opportunity for junior researchers and trainees in LMICs, including individuals external to the core RAISE teams. Participants found the on-demand, online resources to be convenient; however, accessibility varied by country due to poor internet connectivity. Participants reported satisfaction with the Technical Support Centre coaching and found the tailored protocol and manuscript reviews to be helpful.

### Phase III – Impact of technical support centre

A total of *n* = 21, *n* = 11 and *n* = 14 participants responded to the baseline, 12 and 24 month surveys, respectively. Five participants completed the impact survey at all three time points. Mean scores generally improved over time with respect to knowledge translation self-efficacy (Table 3), research utilization and comfort using evidence. Significant differences were observed in researchers’ self-reported ability to develop an evidence-based knowledge translation intervention between 12 and 24-months (*Z* = −2.93, *p* < 0.05).

**Table 3:** Impact of the Technical Support Program on research self efficacy, utilization and comfort

Outcome	Self-reported measure, ability:*	Baseline [*n* = 22] 12 months [*n* = 11] 24 months [*n* = 15]	Mean [SD**]	Median [95% CI***]	Test of difference between baseline and 12-months	Test of difference between 12-months and 24-months
Self-efficacy in the practice of Knowledge Translation [KT]	Conduct a needs assessment	Baseline 12-months 24-months	5.43 [1.17] 5.27 [0.79] 5.64 [0.63]	5.00 [4.90,5.96] 5.00 [4.74,5.80] 6.00 [5.28,6.01]	z score = −0.35 *p* = 0.73	z score = −1.05 *p* = 0.29
	Adapt research evidence	Baseline 12-months 24-months	5.19 [1.33] 5.27 [0.91] 5.71 [0.61]	5.00 [4.59,5.79] 5.00 [4.67,5.88] 6.00 [5.36,6.07]	z score = −0.84 *p* = 0.93	z score = −1.42 *p* = 0.15
	Identify barriers and facilitators	Baseline 12-months 24-months	5.62 [0.92] 5.18 [0.87] 5.79 [0.89]	6.00 [5.20,6.04] 5.00 [4.59,5.77] 6.00 [5.27,6.30]	z score = −1.33 *p* = 0.19	z score = −1.70 *p* = 0.09
	Develop evidence-based KT intervention	Baseline 12-months 24-months	5.14 [1.01] 4.91 [0.54] 5.86 [0.77]	5.00 [4.68,5.60] 5.00 [4.55,5.27] 6.00 [5.41,6.30]	z score = −0.74 *p* = 0.46	z score = −2.93 ***p* = 0.003**
	Develop strategy for monitoring knowledge use	Baseline 12-months 24-months	5.19 [0.98] 4.73 [0.65] 5.14 [1.01]	5.00 [4.74,5.64] 5.00 [4.29,5.16] 5.50 [4.51,5.78]	z score = −1.25 *p* = 0.21	z score = −1.53 *p* = 0.13
	Develop strategy for evaluating relevant outcomes from knowledge use	Baseline 12-months 24-months	5.14 [0.91] 5.00 [0.63] 5.29 [0.83]	5.00 [4.73,5.56] 5.00 [4.58,5.42] 5.00 [4.81,5.76]	z score = −0.28 *p* = 0.78	z score = −1.32 *p* = 0.19
	Develop strategy for sustaining knowledge use over time	Baseline 12-months 24-months	4.43 [1.36] 4.27 [1.10] 5.14 [1.03]	4.00 [3.81,5.05] 4.00 [3.53,5.01] 5.00 [4.55,5.74]	z score = −0.15 *p* = 0.89	z score = −1.92 *p* = 0.06
Research utilization and comfort with evidence	Use evidence to inform decision-making	Baseline 12-months 24-months	6.10 [0.83] 6.45 [0.93] 6.21 [0.89]	6.00 [5.72,6.47] 7.00 [5.83,7.08] 6.00 [5.70,6.73]	z score = −1.49 *p* = 0.14	z score = −0.94 *p* = 0.35
	Comfortable using evidence to inform practice	Baseline 12-months 24-months	6.33 [0.91] 6.36 [1.03] 6.36 [0.75]	7.00 [5.92,6.75] 7.00 [5.67,7.05] 6.50 [5.93,6.79]	z score = −0.36 *p* = 0.72	z score = −0.40 *p* = 0.70

^*^ Likert scale response options from 1 [I cannot do this at all] to 4 [I may be able to do this] to 7 [I can do this well]

^**^ Standard deviation

^***^ Confidence interval

Bolded text denotes a significant result [two-tail *p* < 0.05]

#### Impact of the technical support centre on research self-efficacy, utilization and comfort

In key informant exit interviews, participants noted that the RAISE project enabled them to apply their theoretical knowledge of knowledge translation and HPSR methods. In addition, RAISE participants reported gaining knowledge and skills to engage diverse stakeholders. Participation in the support program and RAISE project also fostered project management skills among junior research staff. Participants suggested they would have benefited from additional collaboration with other RAISE teams, but recognized this ability was hindered by the pandemic. Some participants suggested that the Technical Support Centre should maintain the network of RAISE teams and establish connections between WHO regional and in-country offices to sustain collaboration.

#### RAISE teams’ experiences with health systems guideline adaptation and implementation

Participants reported a number of barriers and enablers regarding implementation of health systems guidelines in LMICs, and highlighted the impact of COVID-19 on implementation efforts (Table 4). Barriers to implementation included: competing health system structures and stakeholder interests, lack of available data, improper maintenance of field equipment to facilitate valid data collection and lack of training among field staff to collect data and facilitate implementation (Table 4). COVID-19 also presented a number of unforeseen barriers to conducting the RAISE research including: project delays, limited human resources/capacity, difficulty engaging stakeholders virtually and participant fears that study personnel would spread COVID-19. Study teams identified the following enablers to continuing their RAISE research during the pandemic: transitioning to a remote study format, use of a rapid analysis approach to facilitate qualitative analysis and leveraging health system stakeholders’ willingness to support research during the pandemic. Enablers included: use of an integrated knowledge translation approach, including inclusion of community members and knowledge users as members of the project team. Teams reported that this approach facilitated buy-in from health systems stakeholders and sustained collaborations during the pandemic. Notably, participants observed a positive impact when stakeholders were engaged during methods development and implementation phases, rather than at study end when recommendations are highlighted. Other enablers included prioritizing diversity in the types of stakeholders engaged in the implementation process (e.g., with respect to occupation, organization), allocation of financial resources to facilitate data collection and partnership strengthening, use of a multi-sectoral approach to facilitate implementation and stakeholder beliefs that the guideline will positively impact health outcomes. Support from the Technical Support Centre to strengthen competency and skills of the implementation team seen as an important enabler to conducting RAISE research (Table 4).

**Table 4:** Barriers and enablers to conducting health system guideline implementation in LMICs

Theme	Description of theme	Participant quote [*n* = 9 interviews]
Barriers to implementation		
Competing health system structures	Health system structures [including the tensions between private and public insurance schemes, country context and limited resources] impact ability to implement health systems guidelines	“In our country, there are many differences between settings, between cities, between insurance companies, between health care providers too. For example, in some hospitals…we have complete rehabilitation services and in others we don’t” P19
Competing stakeholder interests	Professionals in different sectors were challenged to reach consensus on what should be included in the health systems guideline	“the stakeholders have competing interests. For example, we have people coming from mental health background, people coming from public health background, we have those who are working with social work services, we have people from police services working with domestic violence units. So, everybody wants their views to be captured in the guideline and it’s just so difficult to have such different views” P25
Lack of available data limited ability to measure implementation impact	The lack of reliable data on patient outcomes within the country impacted the ability to complete desired analyses to measure impact	“We have many problems with the data because in our country, we don’t have data about the outcomes on patients in general.…This is one of our problems because we can’t [compare] the baseline process to the three or six or twelve months after.” P05 and P19
Lack of equipment to facilitate data collection to assess implementation impact	Lack of maintenance and calibration of field equipment impacted validity of collected data to measure implementation impact	“the equipment they use on field, they’re not…there’s no proper maintenance of those equipment or there is no proper calibration of that equipment and hence the [data measures] are improper [and patient care is impacted]” P15
Lack of awareness/skills among providers tasked with implementation	Lack of awareness by health care providers and field staff on the logistics of the programs they are implementing or evaluating	“In some cases, we also got to know that some of the health care providers are not aware about any such program [to implement the health systems guideline] and there needs to be more awareness.” P15
COVID-19 barriers	The COVID-19 pandemic presented a number of unforeseen barriers including: project delays, limited human resources/capacity, difficulty engaging stakeholders virtually and fears of spreading COVID-19	“We also had difficulties in kind of reaching the pregnant women specifically because they kind of feared that someone from outside is coming and they might carry COVID with them." P15 “One particular thing was the COVID, and I know that is for all the countries, but I think that the Ministry of Health concentrated in trying to solve all of the problems with the pandemic, and many of the human resources and financial resources were directed to this and it was difficult” P05 and P19
Enablers to implementation		
Buy-in from health systems stakeholders via an integrated knowledge translation approach	Having health system stakeholder buy in from study onset was a significant facilitator. The buy-in did not just generate support but also had the decision-makers at the table involved in drafting recommendations, discussing challenges, barriers	“their involvement in the study, not just as respondents but also as active participants in the drafting of the recommendations, critically looking at the challenges, barriers, etc.…if they are supportive, they are kind of involved in it, it makes things pretty, not easier I would say, but makes things much more convenient for us to go ahead with the program" P02 "the most important part was we involved the health system, leadership as investigators, so since we define that objective to them in the discussion process, so they felt the ownership of the project. so although they were kind of very busy in COVID related activity, still they kept track of what is going on. they wanted to know more about how the data collection is coming up and what are the findings.” P15
Diversity among stakeholders engaged in implementation process	The diversity of stakeholders providing feedback on guidelines allowed the research team to gain insights that informed implementation processes	“further, they came from different backgrounds, gave us a more broadened view of how [intervention] should look like and that has helped us to have a guideline that we can say…although not yet fully developed, but at this stage is so comprehensive that we touched on every bits and pieces of [intervention] activities that you can imagine” P25
Competency/skills of implementation team	Career diversity of the research team was found to be an asset to inform implementation	“I guess also our team, our staff team…like, we have a different background…so [individual name] is a doctor, we have an anthropologist, [individual is] an economist, we have people from outside. So, that was really good, actually because we developed different brands in our research and strategies and because of COVID, we needed to be really creative about how to adapt for the implementation.” P31 and P36
Community believes that the guideline will have positive health impacts	Communities believed it was important to eliminate the disease/address the health system issue and saw value in guideline implementation	“I think the willingness to just eliminate [health condition] was a good facilitator for us." P11 "basically in the community, people are supportive of [intervention]…so, they need to be informed, but then there is cooperation once informed.” P15
Use of a multi-sectional approach to facilitate implementation	It is important to engage multiple ministries/stakeholders beyond those involved in health to facilitate implementation	“I think the coming in of the multisectoral approach where it’s not just ministry of health, but it’s a ministry of community development, ministry of water and just bringing this whole multisectoral approach, I think is a great facilitator to eliminating [health condition] because it’s not just about sanitation, but it’s also an issue of livelihoods… having these multisectoral approach is definitely very relevant and needed, especially for health systems guidelines to be translated to local context” P11
Adaptability, particularly in light of COVID-19 pandemic challenges	Teams implemented a variety of strategies to continue their work during the pandemic, which included: transitioning to a remote study format, using a rapid analysis approach to analyze qualitative findings, and leveraging health system stakeholders’ willingness to support and invest in research during the pandemic	“Because of COVID, the health system leadership has been very cooperative and we also…we also knew that they had their priorities. So, because of COVID, they haven’t had any issues working with us on this project” P02 “COVID just made us innovative and how to adapt to challenges and how to overcome challenges. So that made us resilient anyway. Should there be another pandemic, and should we work on a project, these are experiences we have learned from COVID.” P25

Where required, details [health conditions, country name, patient type] were retracted to protect confidentiality of RAISE team participants

## Discussion

Despite considerable improvements, there remains a strong need to develop capacity in the use of knowledge translation and HPSR methods to facilitate health systems guidelines adaptation and implementation in LMICs [31]. The RAISE Technical Support Centre model was rooted in an integrated knowledge translation approach, whereby RAISE teams guided the structure, content and delivery of supports. The program builds upon previous capacity-strengthening initiatives developed by our research team, including programs to strengthen capacity among implementation practitioners in LMIC settings [32, 33].

RAISE participants described the support program to be of high quality, as observed via surveys and confirmed in the key informant interviews. Despite high rates of satisfaction with the program, we did not observe statistically significant impacts on RAISE teams’ self-efficacy in the practice of knowledge translation (with the exception of ability to develop a knowledge translation intervention), research utilization or comfort using evidence. Relatively high baseline scores in these competencies (> 4/7), a small sample size, significant turnover among RAISE research staff and limited ability to participate in capacity-strengthening initiatives due the COVID-19 pandemic may explain the lack of statistically significant impact on these measured outcomes.

Our approach to capacity strengthening aligns with best practices in the field. Mirzoev and colleagues developed a framework for conceptualizing HPSR capacity strengthening. The framework underscores the importance of focusing on the priorities identified by LMIC actors, rather than those driven by high income countries and international actors [34]. In contrast to other HPSR capacity strengthening efforts that use a one-size-fits-all or “blueprint” model to strengthen capacity [35], our approach centred the needs and priorities of LMIC-based RAISE teams. Our emphasis on an integrated knowledge translation approach extended beyond our capacity building efforts and was used by RAISE teams in their own projects [16]. In doing so, RAISE teams centred the priorities, needs and voices of the various knowledge users impacted by their projects, including patients, members of the public, clinicians, community leaders and health system decision-makers. Teams consistently highlighted the benefits of this approach and believed it sustained buy-in throughout the project period and led to the development of guidelines that addressed implementation barriers at onset. Teams reported that early and consistent involvement of knowledge users in the guideline development process (i.e., as members of the project team involved in the methods and implementation phases and not just at study end when recommendations were presented) facilitated continued engagement, even in the context of the pandemic. In addition, our methods leveraged the recommendations of a similar WHO-funded program that utilized a Technical Assistance Centre to strengthen capacity of LMIC research teams to conduct knowledge syntheses. The authors recommend use of a co-design process that extends past the needs assessment, involving policymakers throughout the project lifecycle and enabling cross-country learnings – aspects which we incorporated into our support program [36].

Systematic review findings suggest that capacity strengthening initiatives in LMICs are often driven by non-LMIC actors focused on “spotlight” topics [13, 35, 37]. Capacity strengthening is often perceived as a by-product of conducting health systems research in LMICs, as opposed to a primary goal. In response to these gaps, the Alliance prioritized capacity strengthening as a key feature of the RAISE portfolio and invested in the appointment of the Technical Support Centre. Through this program, we facilitated capacity strengthening that was not limited to research leads, but also extended to research staff, trainees and health system policymakers in LMICs. The potential impact of the supports were realized; RAISE participants reported significant impacts of their projects at the patient/community, organizational and systems levels, despite COVID-19 imposed challenges that delayed and altered the realization of health system impacts.

The COVID-19 pandemic necessitated changes to the planned activities of the Technical Support Centre and RAISE project teams. In response to the pandemic, the Alliance provided a no-cost extension to RAISE teams who faced delays in completing their project and in-country capacity-strengthening workshops were pivoted to a virtual format. RAISE teams reported challenges to conducting RAISE research in the context of the pandemic, yet were able to overcome the majority of these challenges by modifying research activities (e.g., moving from in-person to online engagement/data collection, using rapid analysis methods) and by continuing engagement with community and health system partners. This often necessitated RAISE researchers to deprioritize their projects in order to support the pandemic response and meet knowledge users’ pandemic needs. The pandemic provided some unexpected lessons learned, including the benefits of using rapid analysis approaches to guideline development and implementation, learning to maintain stakeholder engagement in a virtual setting and understanding the importance of multi-sectoral engagement (beyond those involved in the health sector) to facilitate health systems guideline implementation.

Some limitations of this study should be noted. Participation in this evaluation study was not a condition of participating in the RAISE program, meaning not all participants completed surveys or interviews. In addition to a small sample size, there was attrition in survey and interview response, which was exacerbated by the pandemic. This may have contributed to a response bias and challenged the validity of the statistical findings. Nonetheless, qualitative data provide useful insights on the Technical Support Centre’s impact and RAISE teams’ experiences. Participants cited capacity challenges to participation, as the pandemic led to significant study team turnover, conflicting priorities and shifting timelines. With respect to the supports accessed, page views for webinar recordings are likely underreported as many teams downloaded the webinars and watched them as a group due to poor internet connectivity. Finally, we used a non-controlled study design and self-reported measures (subject to social desirability bias [38]) to evaluate the impact of the support program and therefore cannot determine a causal relationship between the implemented processes and impact.

## Conclusions

We provide a model for supporting HPSR capacity strengthening among LMIC stakeholders. A key feature of our model is the emphasis of a user-driven approach to determine priorities, needs and support activities delivered. Other key features included the support team’s flexibility and willingness to consistently adapt to evolving stakeholder needs; this facilitated our ability to continue to support the RAISE teams, even when faced with COVID-19 challenges. Finally, our use of multi-modal supports facilitated capacity strengthening that extended beyond the RAISE teams to their networks and extended the reach among other stakeholders and trainees. Future capacity-strengthening initiatives to support HPSR within LMIC health systems can aim to build upon the presented Technical Support Centre model.

## Supplementary Information

Supplementary material 1.

## Data Availability

The data generated for the current study are not publicly available in order to protect confidentiality of the RAISE teams. Aggregate data are available from the corresponding author on reasonable request.

## Abbreviations

CFIR: Consolidated framework for implementation research
HPSR: Health Policy and Systems Research
LMIC: Low- and middle-income country
RAISE: Research to Enhance the Adaptation and Implementation of Health Systems Guidelines
TDF: Theoretical domains framework
WHO: World Health Organization
