# Supplementary material for: Implementation and evaluation of a technical support center for the Research to Enhance the Adaptation and Implementation of Health Systems Guidelines in LMICs [RAISE] initiative
Source: Health Res Policy Syst. 2026 Sep 10;24(Suppl 1):73. doi: 10.1186/s12961-026-01462-9 (PMC13560173; doi:10.1186/s12961-026-01462-9)
Supplement: Supplementary file 1 [file 12961_2026_1462_MOESM1_ESM.docx]

**Appendices**

**Appendix A – Summary of RAISE Projects**

| RAISE team Country |  | Guideline Addressed | Research Objective/Question |
| --- | --- | --- | --- |
| India | | Government of India guidelines for antenatal care – Pradhan Mantri Surakshit Matritva Abhiyan (PMSMA) guidelines on identification and management of pregnancy health conditions and complications | To improve the implementation of the PMSMA program for management of high-risk pregnancies through the assessment of the degree of adaptation, operational processes and challenges involved in implementation of PMSMA guidelines in tribal communities in Pune district. |
| Nigeria | | WHO guideline recommendations on digital interventions (WHO-GRDI) for health systems strengthening | To adapt and conduct a pilot implementation of the WHO-GRDI for mental health systems in Nigeria and identify the feasibility, acceptability, and challenges to implementation of the guideline and identify strategies to overcome such challenges. |
| Mozambique | | The benefit of addressing wait times was highlighted in multiple guidelines, including:   - 2018 Lancet Global Health Commission on waiting times - WHO Operations Manual for Delivery of HIV Prevention, Care and Treatment at Primary Health Centres in High-Prevalence, Resource-Constrained Settings   By examining how to make “reduce waiting time” an adaptable and implementable guideline to achieve high-quality health systems, their study will inform multiple guidelines | Test whether implementation of a scheduling system for patients living with HIV who regularly visit public health centers in Mozambique can reduce their waiting time for care and increase patient and provider satisfaction; Provide evidence regarding the challenges of implementing management reforms; Identify facility characteristics that affect intervention implementation fidelity. |
| Zambia | | Implementing Zambia multi-sectoral cholera elimination plan (MCEP)  Will compare how MCEP aligns with Global Task Force for Cholera Control guidelines (GTFCC) | Use systems thinking to identify and evaluate existing health systems policies and understand the interconnectedness of the factors that influence health systems vital for the uptake and adaptation of the multi-sectoral cholera elimination plan (MCEP) in Zambia.   - Evaluate the MCEP and GTFCC in order to provide recommendations for MCEP implementation - Explore the barriers and facilitators of the implementation of the MCEP - Explore why and how the MCEP evolved without a national Cholera policy - Explore how the resourcing of the various sectors involved under the MCEP affect implementation - Explore how political and administrative structures influence capacities to implement MCEP |
| Ghana | | Reviewing all existing guidelines on home visits and adapt selected guidelines for home visits to be applicable in the Ghanaian context | To review existing guidelines on home visits, select relevant guidelines, engage stakeholders to adapt and validate existing guidelines on home visits to be applicable in Ghana, complete a formative evaluation to prepare for implementation by piloting the adapted guideline in two districts, and develop and execute a plan to implement the adapted guideline. |
| Colombia | | Will engage policy makers and other key stakeholders to prioritize key recommendations for implementation (5 from one of the WHO guidelines on health systems, and 5 from one of the 58 Colombian clinical practice guidelines) | To support policymakers to use evidence to inform the decision-making and implementation of strong evidence-based health systems recommendations in Colombia, and therefore contribute to a change in how policymakers use evidence in the quotidian decisions about guidelines implementation.  The team engaged policymakers and stakeholders to prioritize recommendations for implementation, and developing a consensus plan to implement health system guidelines. |

**Appendix B – Mean rank score for workshop topics across all participants (from topics rated most to least important)**

| Topic | Average Ranking out of 7 Across All Participants (n=21) | Ranking Order (Most to least important) |
| --- | --- | --- |
| Overview of knowledge translation/ implementation methods | 3 | 1 |
| Adapting guidelines to local country and context | 3.74 | 2 |
| Prioritizing guidelines for implementation | 3.74 | 3 |
| Overview of health systems/ policy research methods | 4.05 | 4 |
| Involving the knowledge users in the implementation process (i.e., Gaining buy-in/engagement from stakeholders/policy makers/ministry) | 4.16 | 5 |
| Specific challenges/opportunities relating to implementation in low- and middle- income countries | 4.63 | 6 |
| Taking an organized approach to project implementation (e.g., budget planning, Project timelines, Deliverables, etc.) | 4.68 | 7 |

**Mean rank score for webinar topics across all participants (from topics rated most to least important)**

| Topic | Average Ranking out of 7 Across All Participants (n=21) | Ranking Order (Most to least important) |
| --- | --- | --- |
| Utilizing knowledge translation principles to adapt and implement health systems guidelines | 3 | 1 |
| Identifying barriers and facilitators to guideline implementation | 3.79 | 2 |
| Identifying and engaging stakeholders to create an implementation team | 3.95 | 3 |
| Operationalizing implementation strategies (i.e., what is the plan to implement your RAISE initiative?) | 4.79 | 4 |
| Assessing and tailoring interventions to organizational and country context | 4.84 | 5 |
| Qualitative and mixed-method analysis | 6.37 | 6 |
| Planning and conducting a process and outcome evaluation | 7.11 | 7 |
| Planning for program scale up | 7.37 | 8 |
| Planning for program sustainability | 7.68 | 9 |
| Preparing a manuscript for publication | 8.63 | 10 |
| Preparing and amending a local or global Institutional Review Board application (i.e., ethics application) | 9.79 | 11 |
| Q14-12: End of project knowledge translation (i.e. dissemination of project findings) | 10.68 | 12 |

## **Appendix C – Webinar series reach analysis (Canvas page views)**

|  | Webinar title | Average webinar page views | Average discussion board page views | Average resource page views |
| --- | --- | --- | --- | --- |
| Webinar 1 | Introduction to RAISE for Research Staff | 10 | 13 | 1.8 |
| Webinar 2 | The AGREE Approach to guideline development, evaluation, and adaptation | 22 | 14.5 | 9 |
| Webinar 3 | Guideline adaptation | 13 | 8 | 2.47 |
| Webinar 4 | Engaging patients | 10.33 | 7.33 | 1.04 |
|  | Engaging healthcare providers |  |  |  |
|  | Engaging decision makers |  |  |  |
| Webinar 5 | Qualitative research | 6 | 0 | 3 |
|  | Rapid qualitative analysis |  |  |  |
| Webinar 6 | Unique KT considerations for LMICs | 0 | 3 | 1.17 |
| Webinar 7 | Project management and administration | 6 | 0 | 1.25 |
|  |  |  |  |  |
| Mean | | 9.62 | 6.5 | 2.82 |
| Median | | 10 | 7.33 | 1.8 |
| Mode | | 6 | 0 | N/A |
| Range | | 22 | 14.5 | 7.96 |
| Standard Deviation | | 6.88 | 5.85 | 2.82 |
